# Isolation of dengue virus serotype 4 genotype II from a patient with high viral load and a mixed Th1/Th17 inflammatory cytokine profile in South Brazil

**DOI:** 10.1186/s12985-016-0548-9

**Published:** 2016-06-06

**Authors:** Diogo Kuczera, Lorena Bavia, Ana Luiza Pamplona Mosimann, Andrea Cristine Koishi, Giovanny Augusto Camacho Antevere Mazzarotto, Mateus Nóbrega Aoki, Ana Maria Ferrari Mansano, Ediléia Inês Tomeleri, Wilson Liuti Costa Junior, Milena Menegazzo Miranda, Maria Lo Sarzi, Wander Rogério Pavanelli, Ivete Conchon-Costa, Claudia Nunes Duarte dos Santos, Juliano Bordignon

**Affiliations:** Laboratório de Virologia Molecular, Instituto Carlos Chagas, Fiocruz, Curitiba, PR Brazil; Secretaria Municipal de Saúde de Cambé, Cambé, PR Brazil; Laboratório de Imunoparasitologia Experimental, Universidade Estadual de Londrina, Londrina, PR Brazil

**Keywords:** Dengue virus serotype 4, Paraná State, Genetic characterization, Cytokines, IL-6, IFN-gamma, IL-17, Viremia

## Abstract

**Background:**

We report the isolation and characterization of dengue virus (DENV) serotype 4 from a resident of Santa Fé, state of Paraná, South Brazil, in March 2013. This patient presented with hemorrhagic manifestations, high viral load and, interestingly, a mixed Th1/Th17 cytokine profile.

**Case presentation:**

The patient presented with classical dengue symptoms, such as fever, rash, myalgia, arthralgia, and hemorrhagic manifestations including petechiae, gum bleeding and a positive tourniquet test result. A serum sample obtained 1 day after the initial appearance of clinical symptoms was positive for NS1 viral antigen, but this sample was negative for both IgM and IgG against DENV. Dengue virus infection was confirmed by isolation of the virus from C6/36 cells, and dengue virus serotyping was performed via one-step RT-PCR. The infection was confirmed to be caused by a serotype 4 dengue virus. Additionally, based on multiple alignment and phylogeny analyses of its complete genome sequence, the viral strain was classified as genotype II (termed LRV13/422). Moreover, a mixed Th1/Th17 cytokine profile was detected in the patient’s serum, and this result demonstrated significant inflammation. Biological characterization of the virus via in vitro assays comparing LRV13/422 with a laboratory-adapted reference strain of dengue virus serotype 4 (TVP/360) showed that LRV13/422 infects both vertebrate and invertebrate cell lines more efficiently than TVP/360. However, LRV13/422 was unable to inhibit type I interferon responses, as suggested by the results obtained for other dengue virus strains. Furthermore, LRV13/422 is the first completely sequenced serotype 4 dengue virus isolated in South Brazil.

**Conclusion:**

The high viral load and mixed Th1/Th17 cytokine profile observed in the patient’s serum could have implications for the development of the hemorrhagic signs observed, and these potential relationships can now be further studied using suitable animal models and/or in vitro systems.

**Electronic supplementary material:**

The online version of this article (doi:10.1186/s12985-016-0548-9) contains supplementary material, which is available to authorized users.

## Background

Dengue virus serotype 4 (DENV-4) genotype II first circulated in Brazil in 1981 and 1982 in limited outbreaks in Boa Vista (Roraima), North Brazil [1], though no other case related to this serotype was reported in the next 25 years. The resurgence of DENV-4 was reported in Manaus (the Amazon region of Brazil) in 2008, when the virus was isolated from serum samples of three patients with no recent travel history [2]. Phylogenetic analyses indicated that these viruses belong to genotype I, which were circulating in Asian countries but not in the Americas [3]. In 2010, the reemergence of DENV-4 was officially recognized by the Brazilian Ministry of Health when it was again notified cases in the northern region of Brazil (Boa Vista, Roraima) [4]. The strains isolated at that time were classified as genotype II [5]. Currently, dengue virus in Brazil is considered endemic, and four dengue virus serotypes are co-circulating [6]. Here, we describe a non-fatal clinical case of dengue virus from a patient with high viral load and a mixed Th1/Th17 cytokine profile. Additionally, phylogenetic analyses and in vitro biological characterization of the new virus strain were performed**.**

## Case presentation

This study addressed a 45-year-old Caucasian man who was a resident of the county of Santa Fé (23° 2′ 16″ S, 51° 48′ 18″ W), located at the North region of the state of Paraná, in South Brazil. The patient presented the first signs and symptoms of dengue virus infection at Cambé (23° 16′ 33″ S, 51° 16′ 40″ W), a city 87.1 km from Santa Fé (Additional file 1: Figure S1). The patient presented at a public health system unit on March 20, 2013, 1 day after the beginning of symptoms, which included fever, rash, myalgia, arthralgia, diarrhea, prostration, itch, retro-orbital pain, nausea, petechiae and gum bleeding. Additionally, the tourniquet test assessing capillary fragility produced positive results. A peripheral blood sample was collected after the patient’s consent with the approval of the FIOCRUZ Research Ethics Committee (n°. 617/11). The patient’s serum tested positive for NS1 antigen (PanBio, QLD, Australia) but negative for antibodies IgM (PanBio, QLD, Australia) and IgG (PanBio, QLD, Australia) against dengue virus.

### Virus isolation and serotyping

**Fig. 1:**
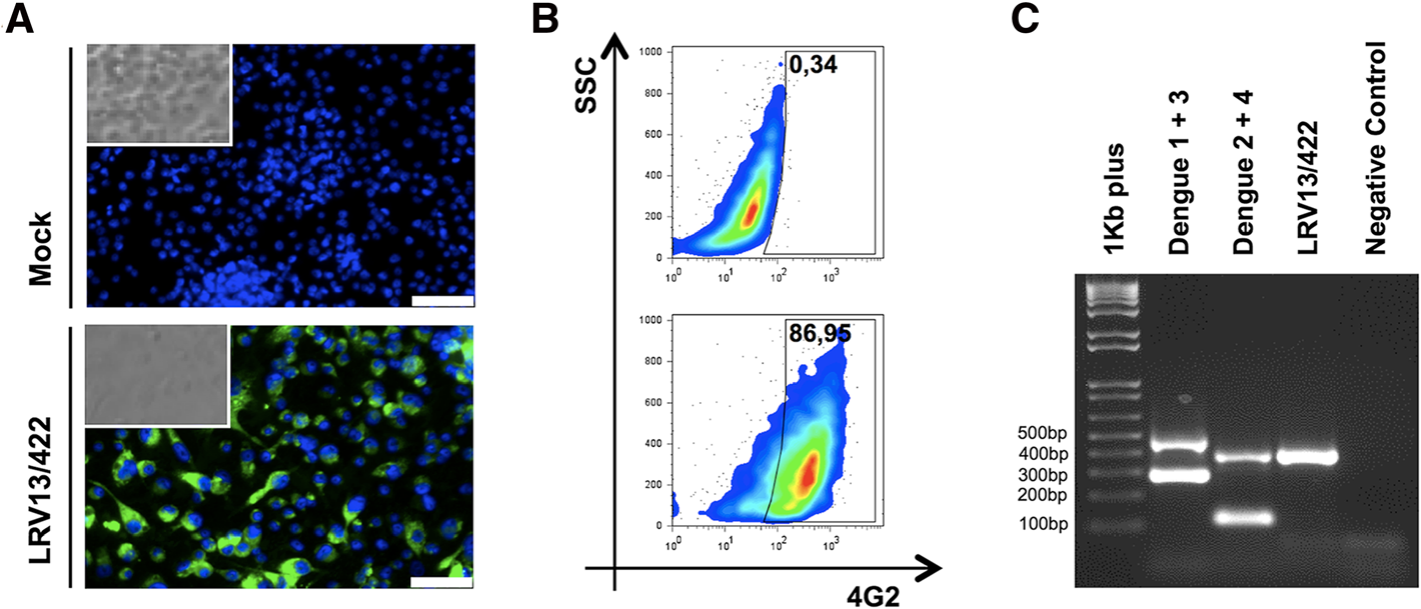
Virus isolation in the C6/36 cell line. Indirect immunofluorescence of C6/36 cells uninfected (mock) or infected for 1 h with patient serum previously diluted (10-fold) in L-15 (Leibovitz) medium and filtered (0.22 μm). After 10 days, the cells were fixed in methanol and stained with a mouse monoclonal antibody against flavivirus E protein (4G2) followed by Alexa-Fluor 488-conjugated anti-mouse immunoglobulin. Then, the cells were observed under a fluorescence microscope. The scale bar corresponds to 50 μm (**a**). Infection was also evaluated via FACS (**b**), and the virus serotype was defined via one-step RT-PCR (**c**). The PCR amplification product was analyzed after electrophoresis in a 1 % agarose gel and staining with ethidium bromide. As RT-PCR positive controls, we used mixtures of DENV-1 and DENV-3 (482 and 290 bp, respectively) as well as DENV-2 and DENV-4 RNAs (119 and 392 bp, respectively)

The patient’s serum was diluted (1:10) in L-15 medium containing gentamicin (25 μg/mL) and 0.26 % tryptose, filtered at 0.22 μm and inoculated into C6/36 *Aedes albopictus* cells (ATCC CRL-1660; 500 μL of 1:10 serum per 25 cm^2^ cell culture flask containing 2.0 × 10^6^ cells or 200 μL of 1:10 serum per 24-well plate containing 1.0 × 10^5^ cells/well). After incubation for 45–60 min at 28 °C, the inoculum was removed. Cells that had ultimately detached (due to the action of complement system factors present in the human serum sample) were recovered via centrifugation and resuspended in L-15 media containing gentamicin (25 μg/mL), 0.26 % tryptose and 5 % fetal bovine serum (FBS). Then, the cells were added to the same cell culture flask (25 cm^2^) or 24-well plate. The cells were cultured at 28 °C for 10 days. C6/36 cells in 24-well plates were washed once with 1× PBS and then fixed and permeabilized with 1:1 (v/v) ethanol:acetone solution at -20 °C for 1 h. The monoclonal anti-flavivirus E protein 4G2 antibody (1:100 in 1× PBS) [7] was used to stain DENV-infected cells for 45 min at 37 °C. Cells were washed and incubated with an Alexa 488-conjugated anti-mouse secondary antibody (1:400 in 1× PBS) (Life Technologies), and nuclei were stained with DAPI (300 nM). Finally, images were captured with a fluorescence microscope (Leica DMI6000B) using LAS AF (Leica) software. The immunofluorescence assay (IFA) showed that a high percentage of C6/36 cells was positive for dengue virus (Fig. 1a). Furthermore, C6/36 cells were detached from the 25 cm^2^ cell culture flask, and the percentage of infected cells was quantified via a fluorescence-activated cell sorting (FACS) assay, as described previously [8], using a FACSCANTO II Flow Cytometer (BD). The FACS assay showed that more than 85 % of the C6/36 cells were stained with 4G2 antibodies (Fig. 1b). Both the IFA and FACS assays confirmed dengue virus isolation from serum samples.

To serotype the dengue virus and confirm IFA results, RNA from cell culture (C6/36) supernatant was extracted using a QIAamp Viral RNA Mini Kit (QIAGEN, Hilden, NRW, Germany) as described by the manufacturer. RNA was used as a template for one step RT-PCR (QIAGEN) employing the forward primer D1 and reverse primers TS1, TS2, TS3 and TS4 (Table 1) adapted from a protocol previously described [9]. The following cycle was used for amplification: 50 °C for 30 min, 95 °C for 15 min, followed by 35 cycles of 95 °C for 30 s, 57 °C for 45 s and 72 °C for 1 min, and finally 72 °C for 10 min. The amplification product was analyzed by electrophoresis in 1 % agarose gels stained with ethidium bromide. A mixture of RNA from DENV-1 and -3 (DENV-1 BR/90 [GenBank: AF226685.2] and DENV-3 BR/97-04 [GenBank: EF629367.1]) and DENV-2 and -4 (DENV-2 ICC/266 and DENV-4 TVP/360 [GenBank: KU513442]) were used as positive controls. The one step RT-PCR confirmed dengue virus infection and allowed its classification as serotype 4 (Fig. 1c).

**Table 1:** Primer sequences used for each dengue virus serotype detection

Primer name	Primer sequence (5′-3′)	Genome position (GenBank accession number)
D1	TCAATATGCTGAAACGCGCGAGAAACCG	132-159 (NC_001477 e NC_001475)
		134-161 (NC_001474)
		136-163 (NC_002640)
TS1	GGTCTCAGTGATCCGGGGG	595-613 (NC_001477)
TS2	CGCCACAAGGGCCATGAACAG	232-252 (NC_001474)
TS3	TAACATCATCATGAGACAGAGC	400-421 (NC_001475)
TS4	CTCTGTTGTCTTAAACAAGAGA	506-527 (NC_002640)

### Patient viremia and serum cytokine profile

**Fig. 2:**
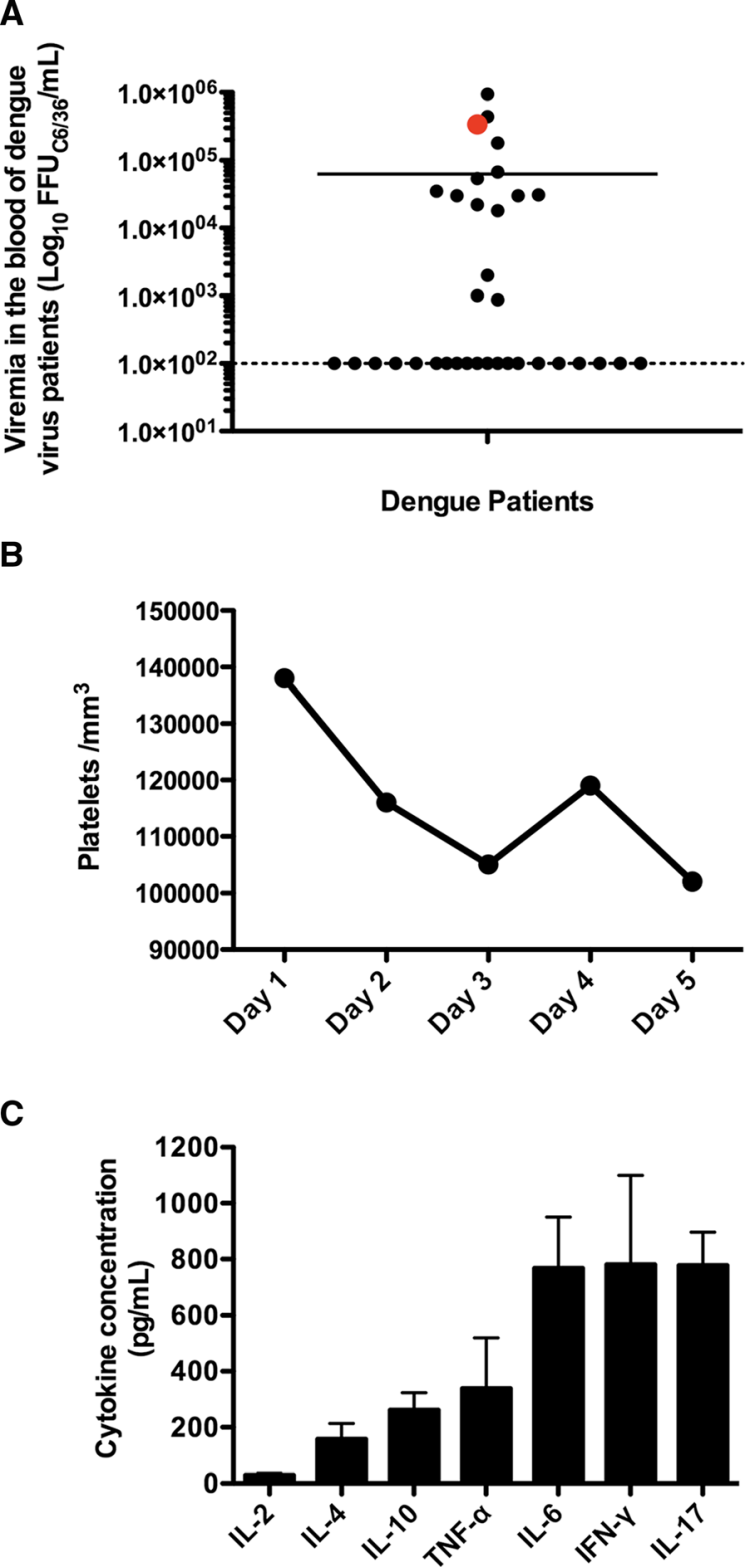
Patient viral load, platelet count and serum cytokine concentrations. Viral load in the patient’s serum sample was measured with a focus-forming assay (using 4G2 antibody) in C6/36 cells 1 day after the beginning of the symptoms (**a**). The viral load in the patient’s serum (*red dot*) was compared with that in samples from 34 other patients with acute-phase DENV-1 infection (*black dots*; the detection limit was 100 FFU_C6/36_/mL). Platelet counts (reference values between 150,000 and 450,000/mm^3^) were monitored for 5 days to assess the risk of severe hemorrhage (**b**). Additionally, the levels of a T helper cytokine profile (IL-2, IL-4, IL-6, IL-10, IL-17A, IFN-γ and TNF-α) were measured in the patient’s serum (1 day after the symptoms appeared) using a Cytometric Bead Array (CBA; Becton & Dickinson). A CBA assay was performed in three technical replicates, and the values represent the means ± SEM (**c**)

Considering the large number of infected cells detected in the virus isolation (Fig. 1a and b), the patient’s symptoms and the potential role of the inflammatory response in dengue virus infection, we evaluated the patient’s viral load and T helper cytokine profile in the serum sample. The patient displayed a viral load of 3.4 × 10^5^ FFU_C6/36_/mL based on titration of the patient’s serum in a focus-forming assay in the *Aedes albopictus* cell line C6/36, which was adapted from a previously described protocol [10]. Comparing the viral load of this patient with that of 34 other patients acutely infected with dengue virus serotype 1 (Cambé, Paraná, Brazil) provided evidence of a high viral load in the blood of this patient (Fig. 2a). Furthermore, analyses of platelet counts during the five initial days showed values between 102,000 and 138,000 platelets/mm^3^, which confirmed the presence of thrombocytopenia considering the reference values (between 150,000 and 450,000 platelets/mm^3^) (Fig. 2b). It is known that high viral load and NS1 levels early in the course of dengue virus infection are positively associated with hospitalization length and negatively associated with platelet count [11].

In addition to the viral load, the T helper response plays a role in the manifestation of hemorrhage in association with dengue virus infection [12]. Because the patient presented with hemorrhagic symptoms (petechiae and gum bleeding), thrombocytopenia and a high viral load, we decided to evaluate the T helper cytokine profile using a human Th1/Th2/Th17 BD™ Cytometric Bead Array kit (BD Biosciences, San Diego, CA, USA) according to the manufacturer’s recommendations. The data demonstrated low levels of IL-2 and the secretion of IL-4, IL-10 and TNF-α. However, the presence of high levels of IFN-γ, IL-17A and IL-6 (compared to the levels of these cytokines in adult healthy donors) [13] suggested that a mixed Th1/Th17 cytokine response could play a role in both dengue virus replication control and hemorrhagic manifestations (Fig. 2c). A Th1-type response has been observed more frequently in dengue fever (DF) patients than in patients with severe dengue virus infection [12]. Additionally, IFN-γ, a Th1 cytokine, was essential for DENV replication control and host resistance in a mouse model, and these results indicated a protective role of this cytokine during dengue virus infection [14]. However, it was shown that high levels of cytokines produced by T cells, macrophages/monocytes and endothelial cells (TNF-α, IFN-γ, IL-10, IL-6 and IL-8) contributed to endothelial cell damage and hemorrhagic manifestations due to a phenomenon known as a cytokine storm [14–16]. Additionally, in dengue-infected patients, the IFN-γ levels were increased in severe cases compared to cases of mild disease [17]. Moreover, treatment with anti-TNF-α antibodies significantly reduced mortality in a mouse model of lethal dengue virus serotype 2 infection, thus reinforcing the role of cytokines in dengue pathogenesis [18].

Furthermore, the role of IL-17 in the severity of dengue fever has not been well elucidated. Arias and colleagues showed that an increase in IL-17 expression was not associated with the severity of disease, primary or secondary infection or DENV serotype [15]. On the other hand, Jain and colleagues demonstrated that high levels of IL-17 were associated with severe dengue infection compared to dengue without warning signs [19]. Despite the observation of a high level of IL-17 in the patient’s serum, the effect of IL-17 on disease severity must be evaluated using a larger number of samples before concluding that this cytokine plays a protective or pathogenic role in dengue virus infection. Additionally, many pro-inflammatory mediators are produced in response to IL-17, such as IL-6 and neutrophil- and granulocyte-attracting chemokines, and the levels of these factors are increased during DENV infection [20, 21].

High levels of IL-6 have been related to dengue pathogenesis and hemorrhagic symptoms and have been established as a biomarker of DENV infection [15, 22, 23]. This cytokine mediates the increases in endothelial cell permeability and in the production of anti-platelet or anti-endothelial cell autoantibodies, leading to plasma leakage and bleeding [23, 24].

In this fashion, a mixed cytokine profile (Th1/Th17) might induce an important and protective (controlling virus replication) pro-inflammatory response. However, a function of secreted cytokines in promoting the development of the hemorrhagic manifestations observed in the patient cannot be ruled out [14–16].

### In vitro characterization

**Fig. 3:**
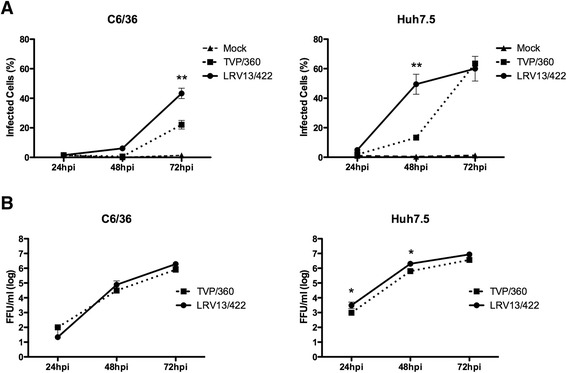
DENV-4 LRV13/422 infection profile in vitro. C6/36 and Huh7.5 cells were seeded in 96-well plates (1 × 10^4^ cells/well) and were infected with DENV-4 (TVP/360 and LRV13/422) at a MOI of 0.1 in Leibovitz (L-15) or DMEM/F12 medium, respectively, without FBS for 1 h and 30 min. The inocula were then removed, and medium supplemented with 5 % (C6/36) or 10 % (Huh7.5) FBS in a final volume of 200 μL. After 24 h, 48 h or 72 h post-infection, cells were fixed and permeabilized, and the extent of infection was analyzed in an Operetta system using 4G2 primary antibody and an Alexa 633-conjugated secondary antibody (**a**). Additionally, viral titration of C6/36 and Huh7.5 cell supernatants was performed to analyze the secretion of viable viral particles (**b**). Data were analyzed using a two-way ANOVA followed by Bonferroni’s test. Values represent the means ± SD of the results of three independent experiments. * *P <*0.05; ** *P <*0.01

To evaluate the ability of the isolated virus (LRV13/422) to infect vertebrate and invertebrate cells, a kinetic assay was performed using C6/36 and human hepatoma cell lines (Huh7.5; ATCC PTA-8561), and LRV13/422 was compared with the DENV-4 reference strain TVP/360. Both C6/36 and Huh7.5 cells (1 × 10^4^ cells/well in 96-well plates) were infected at a MOI of 0.1 in the respective culture medium (L-15 or DMEM/F12 medium) without FBS for 1 h 30 min. Then, the inoculum was removed, and complete medium was added. At 24, 48 and 72 h post-infection (hpi), cells were fixed and permeabilized with methanol:acetone (v/v), and cell infection was analyzed in the Operetta High-Content Imaging System (PerkinElmer) using 4G2 primary antibodies and Alexa 633-conjugated secondary antibodies. The high-content imaging system provides the quantification and an image of dengue virus-infected cells, representing an advantage compared to traditional FACS. The data demonstrated that LRV13/422 displayed higher infection ability than TVP/360 in both C6/36 (72 hpi) and Huh7.5 cells (48 hpi) (Fig. 3a; Additional file 2: Figure S2). However, titration of the cell culture supernatant confirmed only the higher replication of LRV13/422 in Huh7.5 cells (24 and 48 hpi) compared to TVP/360 (Fig. 3b). On the other hand, TVP/360, a cell culture-adapted dengue virus strain, has previously been shown to replicate more rapidly than other clinical isolates [25].

**Fig. 4:**
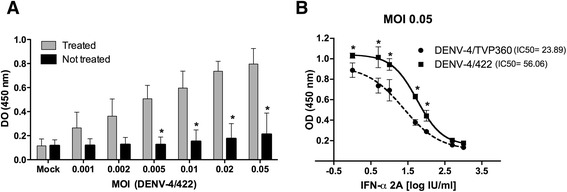
Response to type I IFN treatment. Huh7.5 cells were infected with DENV-4 TVP/360 or LRV13/422. After infection at different MOIs (from 0.001 to 0.05), the cells were treated with 100 IU/mL IFN-α 2A. * *P <*0.05 compared to non-treated controls (**a**). Additionally, Huh7.5 cells were infected at a MOI of 0.05 and treated with a range of IFN-α 2A concentrations (from 1 to 1000 IU/mL). For the IFN-α concentration response curve data were analyzed using a sigmoidal dose-response curve (variable slope). * *P <*0.05 between strains (**b**). Data were analyzed using a two-way ANOVA followed by Bonferroni’s test, and represent the means ± SD of four independent experiments

Additionally, to evaluate the ability of DENV-4 LRV13/422 to circumvent type I IFN antiviral activities in comparison with the adapted DENV-4 strain (TVP/360), Huh7.5 cells were seeded at a density of 2 × 10^4^ cells/well on a flat-bottom 96-well plate 16 h prior to infection. Then, the medium was removed, and 100 μL of viral inocula of DENV-4 TVP/360 or DENV-4 LRV13/422 (MOI of 0.001 to 0.05) were added to the wells. After incubation for 1 h 30 min (at 37 °C in 5 % CO_2_), the inoculum was replaced with medium (200 μL) or medium containing 100 IU of IFN-α 2A (Blausiegel). After 72 h, an *in situ* ELISA was performed as described by Koishi and colleagues to quantify the dengue virus E antigen concentration as a surrogate of virus replication [25]. The E protein ELISA system has been shown to be an easy and rapid technique to quantify dengue virus antigen levels in cell culture [25]. The E protein ELISA was validated for antiviral screening compared with titration via focus forming assays and NS1 secretion measurements [25]. The results showed that type I IFN was able to control LRV13/422 replication at the MOI tested (Fig. 4a). This result suggested that LRV13/422 was not able to inhibit type I IFN. It was shown that dengue virus inhibits type I IFN responses in human cells in a strain-dependent manner [26]. The ability to inhibit type I IFN responses suggest the existence of strain-specific virulence factors that are able to assist dengue virus in escaping from innate immune responses [26]. Additionally, the sensitivity of both LRV13/422 and TVP/360 to type I IFN was analyzed using a concentration-response curve of IFN-α 2A (1 to 1000 IU/ml) and an *in situ* ELISA (Fig. 4b and c) [25]. Notably, the IC_50_ value for LVR13/422 was higher than that for the control TVP/360 (Fig. 4c).

### Sequencing and phylogenetic analyses

**Fig. 5:**
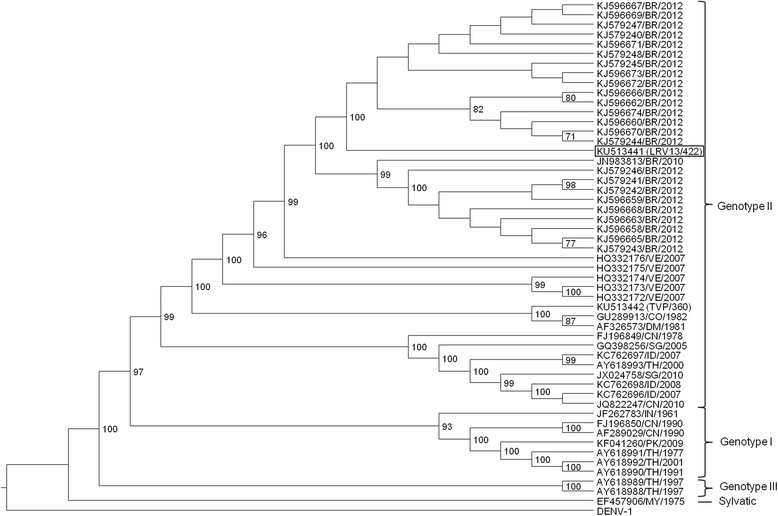
Phylogenetic tree of dengue virus serotype 4. Phylogenetic analysis based on the complete nucleic acid sequence of a 2013 Brazilian isolate of DENV-4 (LRV13/422). The phylogenetic tree was inferred using the maximum likelihood algorithm based on the general time-reversible model with gamma-distributed rate variation and a proportion of invariable sites (GTR+I+G), as implemented in MEGA6.06. The numbers shown to the right of the nodes represent bootstrap support values >70 (1000 replicates). The tree was rooted with DENV-1 (NC_001477.1), and the branch lengths do not represent the genetic distance. The strains are labeled according to GenBank accession number/2-letter country code/year of isolation

The entire genome sequence of DENV-4 strain LRV13/422 was determined and used for genetic characterization. RNA (from C6/36 cell culture supernatants obtained after virus isolation from the patient’s serum - passage 0) was reverse-transcribed using RT-Improm II (Promega, Madison, WI, USA) and random primers (12.5 pmol/μL) according to the manufacturer’s protocols. The resulting cDNA was amplified via PCR using a high-fidelity enzyme (QIAGEN LongRange PCR Kit, Hilden, NRW, Germany) and specific primers (Additional file 3: Table S1). The purified PCR products were sequenced by Macrogen Inc. (Seoul, Korea). To sequence the genome extremities, RNA was de-capped (Tobacco Acid Pyrophosphatase, Epicentre, Madison, WI, USA), ligated (RNA ligase, New England Biolabs, Ipswich, MA, USA) and subjected to RT-PCR using the primers D4.23 and TS4 (Additional file 3: Table S1 and Table 1, respectively). The sequences were assembled using the phred/Phrap/consed software package (www.phrap.org) [27–30]. The final consensus sequence of LRV13/422 was deposited in GenBank under accession number KU513441. The phylogenetic analysis results (Fig. 5) placed this new isolate together with other viruses from genotype II. The highest nucleotide identity was observed between LRV13/422 and the DENV-4/MT/BR27_TVP17913/2012 strain (GenBank: KJ579247), which was also obtained from Brazil. Pairwise comparison of these strains showed 13 nucleotide differences and 1 amino acid difference, which are detailed in Additional file 4: Table S2. After completion of the in vitro characterization of LRV13/422 in comparison to the reference strain TVP/360, the reference strain was also sequenced (GenBank: KU513442). Differences compared to LRV13/422 are detailed in Additional file 4: Table S2.

## Conclusion

Since the reemergence of DENV-4 in 2010 in Brazil, co-circulation of four dengue virus serotypes has occurred. Additionally, the hyperendemicity of dengue virus observed in Brazil enhanced the severity of the disease and the mortality rate of infection. The isolation of a DENV-4 genotype II in South Brazil five years after entrance of the virus into the northern region of the country is relevant to understanding the spread and epidemiology of dengue virus. Moreover, isolation of the LRV13/422 strain will be useful for studying host-pathogen interactions, diagnosis, immune responses and antiviral development.

## Abbreviations

°C, celsius; μL, microliter; DENV, dengue virus; DF, dengue fever; dpi, days post-infection; E, envelope; ELISA, enzyme-linked immunosorbent assay; FACS, fluorescence-activated cell sorting; FBS, fetal bovine serum; FFU, focus-forming units; h, hour(s); hpi, hours post-infection; IFA, immunofluorescence assay; IFN, interferon; IgG, immunoglobulin G; IgM, immunoglobulin M; IL, interleukin; IU, international unit; MOI, multiplicity of infection; NS1, non-structural protein 1; PBS, phosphate-buffered saline; RT-PCR, reverse-transcription polymerase chain reaction; S, South; Th, T helper; TNF, tumor necrosis factor; v/v, volume/volume; W, West

## Additional files

Additional file 1: Figure S1. Geographical locations of the cities of Santa Fé and Cambé in the state of Paraná, South Brazil. The location of Paraná in Brazil is shown in red. Additionally, Santa Fé (23° 2′ 16″ S, 51° 48′ 18″ W; green) and Cambé (23° 16′ 33″ S, 51° 16′ 40″ W; yellow) are marked in the map. The distance between these two cities is 87.1 km. (TIF 1532 kb) Additional file 2: Figure S2. Kinetics of LRV13/422 infection in C6/36 and Huh7.5 cells. Both C6/36 and Huh7.5 cells were infected with DENV-4 serotypes (TVP/360 and LRV13/422) at a MOI of 0.1. The extent of infection (detected using 4G2 primary antibody and an Alexa 633-conjugated secondary antibody) was analyzed using an Operetta high-content imaging system (PerkinElmer). Images show the significantly different extent of infection of C6/36 (72 hpi) and Huh7.5 (48 hpi) cells between LRV13/422 and TVP/360. (TIFF 9991 kb) Additional file 3: Table S1. Primer sequences for whole genome amplification and sequencing. (DOCX 82 kb) Additional file 4: Table S2. Observed sequence differences among DENV-4/MT/BR27_TVP17913/2012 strain, LRV13/422 and TVP/360-P4. The grey shading indicates the non-synonymous mutations. (DOCX 125 kb)
