# Supplementary figures and images for: Isolation of dengue virus serotype 4 genotype II from a patient with high viral load and a mixed Th1/Th17 inflammatory cytokine profile in South Brazil
Source: Virol J. 2016 Jun 6;13:93. doi: 10.1186/s12985-016-0548-9 (PMC4895951; doi:10.1186/s12985-016-0548-9)

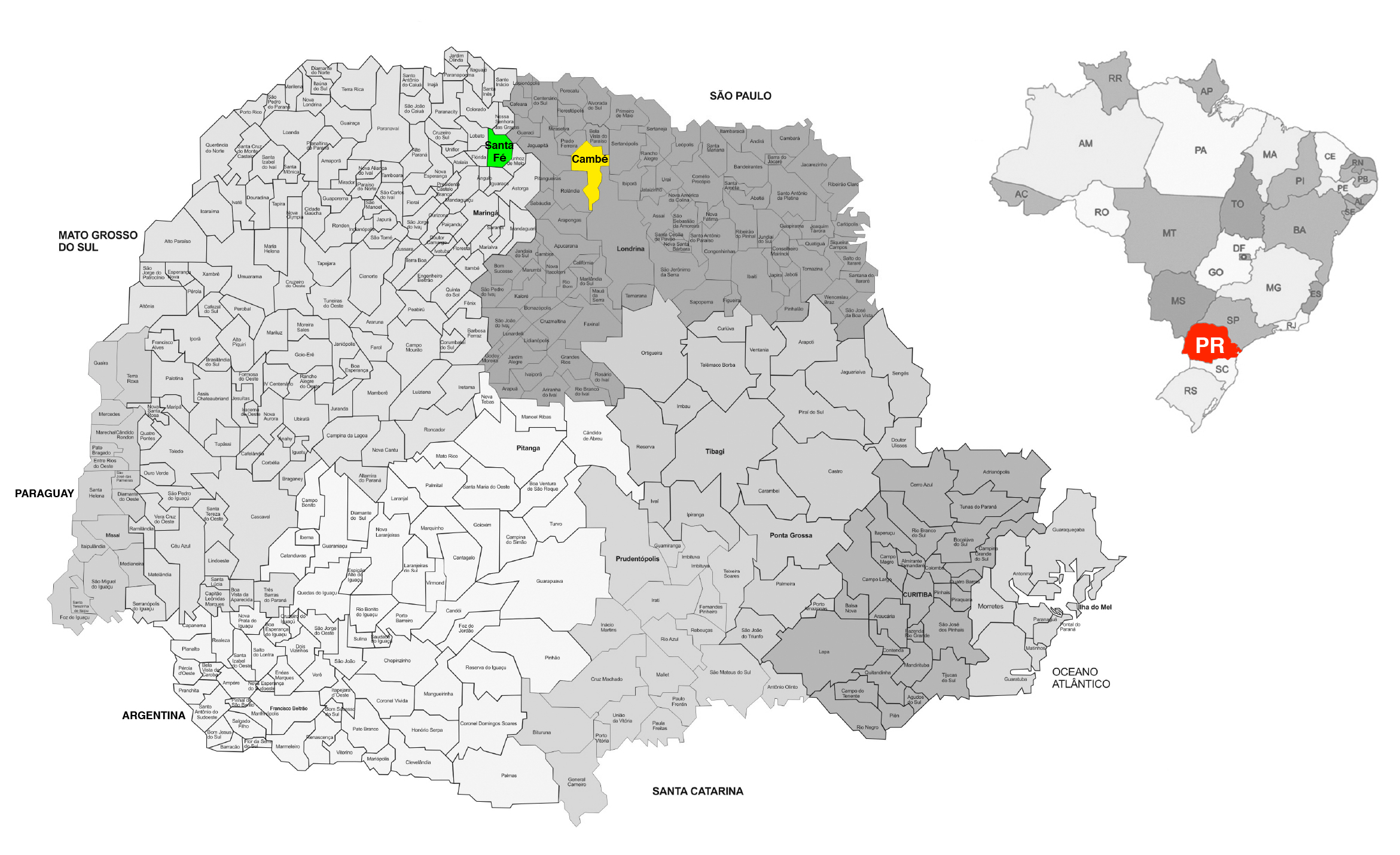

Supplement: Additional file 1: Figure S1. — Geographical locations of the cities of Santa Fé and Cambé in the state of Paraná, South Brazil. The location of Paraná in Brazil is shown in red. Additionally, Santa Fé (23° 2′ 16″ S, 51° 48′ 18″ W; green) and Cambé (23° 16′ 33″ S, 51° 16′ 40″ W; yellow) are marked in the map. The distance between these two cities is 87.1 km. (TIF 1532 kb) [file 12985_2016_548_MOESM1_ESM.tif]

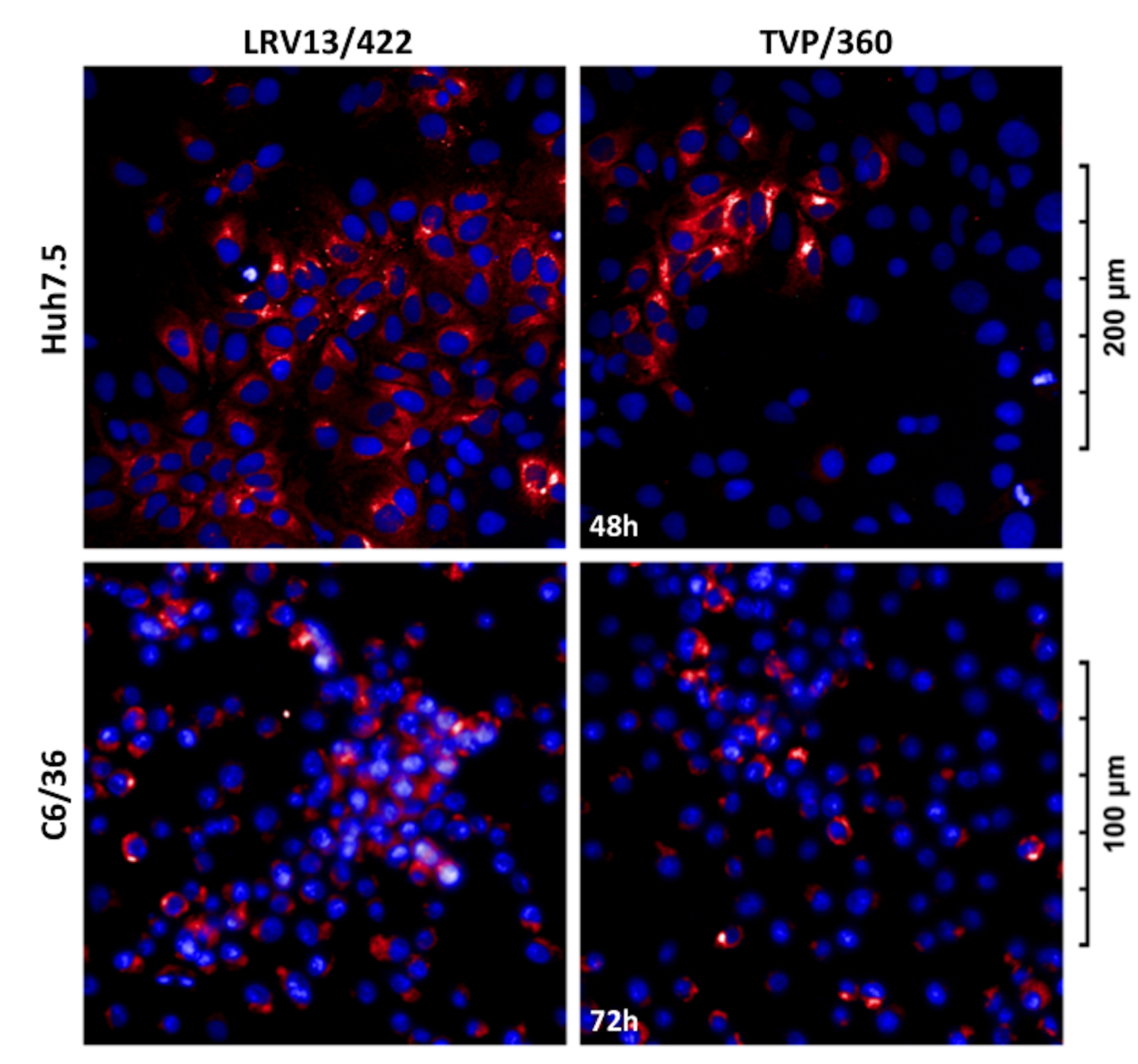

Supplement: Additional file 2: Figure S2. — Kinetics of LRV13/422 infection in C6/36 and Huh7.5 cells. Both C6/36 and Huh7.5 cells were infected with DENV-4 serotypes (TVP/360 and LRV13/422) at a MOI of 0.1. The extent of infection (detected using 4G2 primary antibody and an Alexa 633-conjugated secondary antibody) was analyzed using an Operetta high-content imaging system (PerkinElmer). Images show the significantly different extent of infection of C6/36 (72 hpi) and Huh7.5 (48 hpi) cells between LRV13/422 and TVP/360. (TIFF 9991 kb) [file 12985_2016_548_MOESM2_ESM.tiff]
