# Supplementary material for: Isolation of dengue virus serotype 4 genotype II from a patient with high viral load and a mixed Th1/Th17 inflammatory cytokine profile in South Brazil
Source: Virol J. 2016 Jun 6;13:93. doi: 10.1186/s12985-016-0548-9 (PMC4895951; doi:10.1186/s12985-016-0548-9)
Supplement: Additional file 3: Table S1. — Primer sequences for whole genome amplification and sequencing. (DOCX 82 kb) [file 12985_2016_548_MOESM3_ESM.docx]

**Supplementary Table 1. Primer sequences for whole genome amplification and sequencing.**

| **Primer name** | **Primer sequence (5´ - 3´)** |
| --- | --- |
| D4.1 | GACAAGGACAGTTCCAAATCGGAAGCTTGC |
| D4.2 | CCAATCCATCTTGCGGCGCTCTGTGCCTGG |
| D4.3 | GCAAGCCGAAAGAATTGGAGAGCC |
| D4.4 | CGTAGGGCCTCTTCCATCTCGGC |
| D4.5 | GGGACATGCACCCAGAGCGGA |
| D4.6 | CCCTGGGCCATGGTTGTGACGC |
| D4.7 | GGAGACACCCATGCAGTAGGAAATG |
| D4.8 | CCAACACCTATCACTATGTAGCTGTCCCC |
| D4.9 | GGAATTAGATCAACCACGAGGCTGG |
| D4.10 | GGGTCTTGGGCCACAGACATG |
| D4.11 | GGCAGTGTTCAAGATGTCACCAGG |
| D4.12 | CCAAACCCACAGCCATTATGCC |
| D4.13 | GGAGTGTACAGGATCATGCAAAGAGGG |
| D4.14 | GCTTCTGTCCAGTGGGCATGATCTTCATC |
| D4.15 | GCAACAGCCCAATAGAAGACATCGAG |
| D4.16 | CGTAGATCAATTGATTGTCTTGTGGGGTCC |
| D4.17 | CCAACAACCTTGACAGCATCC |
| D4.18 | CCCAGTGTCTCTCCTGTGGTCCC |
| D4.19 | CCCTGCTCTGTGATATTGGGGAGTC |
| D4.20 | GCAATCGCTGAAGCCTTCTCCC |
| D4.21 | CATGGAGTGGAGTGGAAGGGGAAGG |
| D4.22 | CACCACGCAATCGTCTCCAC |
| D4.23 | GTGTGGAACAGAGTGTGGATAGAAGAC |
| D4.24 | CGCCCAGGTGGCTCTAGAAG |
| D4.25 | GTATACGCTGACCCCATGGC |
| D4.26 | AGAACCTGTTGGATCAACAACACCAATC |
| D4.27 | AGTTGTTAGTCTGTGTGGACCGAC |
| TS4 | CTCTGTTGTCTTAAACAAGAGA |
