# Supplementary material for: Isolation of dengue virus serotype 4 genotype II from a patient with high viral load and a mixed Th1/Th17 inflammatory cytokine profile in South Brazil
Source: Virol J. 2016 Jun 6;13:93. doi: 10.1186/s12985-016-0548-9 (PMC4895951; doi:10.1186/s12985-016-0548-9)
Supplement: Additional file 4: Table S2. — Observed sequence differences among DENV-4/MT/BR27_TVP17913/2012 strain, LRV13/422 and TVP/360-P4. The grey shading indicates the non-synonymous mutations. (DOCX 125 kb) [file 12985_2016_548_MOESM4_ESM.docx]

**Supplementary Table 2:** **Observed sequence differences among DENV-4/MT/BR27_TVP17913/2012 strain, LRV13/422 and TVP/360-P4. The grey shading indicates the non-synonymous mutations.**

| **Genome position (gene)** | **DENV-4/MT/BR27_TVP17913/2012 (KJ579247)^#^** | | **LRV13/422 (KU513441)^#^** | | **TVP/360 (KU513442)^*^** | |
| --- | --- | --- | --- | --- | --- | --- |
|  | **nucleotide** | **aminoacid** | **nucleotide** | **aminoacid** | **nucleotide** | **aminoacid** |
| 73 (5’UTR) | A | - | A | - | G | - |
| 246 (C) | C | L | C | L | T | F |
| 414 (C) | T | L | T | L | C | L |
| 593 (prM) | C | Y | C | Y | T | Y |
| 785 (prM) | A | G | A | G | G | G |
| 839 (prM) | T | F | T | F | C | F |
| 842 (prM) | A | A | A | A | G | A |
| 884 (prM) | G | G | G | G | A | G |
| 1076 (E) | C | T | C | T | T | T |
| 1129 (E) | T | L | T | L | C | S |
| 1217 (E) | T | R | T | R | G | R |
| 1232 (E) | T | D | T | D | C | D |
| 1238 (E) | A | G | A | G | G | G |
| 1295 (E) | C | F | C | F | T | F |
| 1323 (E) | C | L | C | L | T | L |
| 1361 (E) | G | V | G | V | A | V |
| 1415 (E) | G | G | G | G | A | G |
| 1426 (E) | C | T | C | T | T | M |
| 1451 (E) | T | V | T | V | G | V |
| 1461 (E) | C | L | C | L | T | L |
| 1517 (E) | C | F | C | F | T | F |
| 1529 (E) | C | I | C | I | T | I |
| 1532 (E) | A | L | A | L | G | L |
| 1601 (E) | G | T | G | T | A | T |
| 1602 (E) | A | T | A | T | G | A |
| 1613 (E) | T | D | T | D | C | D |
| 1721 (E) | T | H | C | H | T | H |
| 1975 (E) | A | K | G | R | A | K |
| 1988 (E) | C | R | C | R | T | R |
| 1989 (E) | G | V | G | V | A | I |
| 1991 (E) | T |  | T |  | C |  |
| 1998 (E) | G | A | G | A | T | S |
| 2018 (E) | C | N | C | N | T | N |
| 2024 (E) | T | N | T | N | C | N |
| 2097 (E) | C | L | C | L | T | L |
| 2135 (E) | T | G | T | G | C | G |
| 2171 (E) | C | R | C | R | A | R |
| 2180 (E) | C | I | C | I | T | I |
| 2322 (E) | C | L | C | L | T | L |
| 2330 (E) | G | L | A | L | G | L |
| 2366 (E) | C | A | C | A | T | A |
| 2387 (E) | G | G | G | G | A | G |
| 2403 (E) | T | L | T | L | C | L |
| 2414 (E) | G | T | G | T | A | T |
| 2423 (E) | G | A | G | A | A | A |
| 2455 (NS1) | G | R | G | R | A | K |
| 2525 (NS1) | C | F | C | F | T | F |
| 2621 (NS1) | T | V | T | V | C | V |
| 2642 (NS1) | T | N | T | N | C | N |
| 2678 (NS1) | T | D | T | D | C | D |
| 2687 (NS1) | G | V | A | V | A | V |
| 2699 (NS1) | C | D | C | D | T | D |
| 2705 (NS1) | A | K | A | K | G | K |
| 2720 (NS1) | G | K | G | K | A | K |
| 2746 (NS1) | C | A | C | A | T | V |
| 2750 (NS1) | C | S | C | S | T | S |
| 2780 (NS1) | G | G | G | G | A | G |
| 2801 (NS1) | T | P | T | P | A | P |
| 2840 (NS1) | T | D | T | D | C | D |
| 2876 (NS1) | T | N | T | N | C | N |
| 2880 (NS1) | T | F | T | F | C | L |
| 2970 (NS1) | C | L | C | L | T | L |
| 2972 (NS1) | G |  | G |  | A |  |
| 2993 (NS1) | C | D | C | D | T | D |
| 3064 (NS1) | G | R | G | R | A | K |
| 3074 (NS1) | C | L | C | L | T | L |
| 3110 (NS1) | T | H | T | H | C | H |
| 3137 (NS1) | G | E | G | E | A | E |
| 3149 (NS1) | T | L | T | L | C | L |
| 3167 (NS1) | A | A | A | A | G | A |
| 3206 (NS1) | T | A | T | A | C | A |
| 3221 (NS1) | T | G | T | G | C | G |
| 3233 (NS1) | G | L | G | L | A | L |
| 3240 (NS1) | C | L | C | L | T | L |
| 3278 (NS1) | T | V | T | V | C | V |
| 3291 (NS1) | A | N | A | N | G | D |
| 3359 (NS1) | T | C | T | C | C | C |
| 3374 (NS1) | A | T | A | T | G | T |
| 3380 (NS1) | C | P | C | P | T | P |
| 3395 (NS1) | A | L | A | L | G | L |
| 3461 (NS1) | T | V | T | V | C | V |
| 3494 (NS2A) | G | S | G | S | A | S |
| 3503 (NS2A) | C | F | C | F | T | F |
| 3512 (NS2A) | T | G | T | G | C | G |
| 3551 (NS2A) | A | R | A | R | G | R |
| 3563 (NS2A) | C | T | C | T | T | T |
| 3566 (NS2A) | A | R | A | R | G | R |
| 3583 (NS2A) | C | A | C | A | T | V |
| 3590 (NS2A) | A | V | A | V | G | V |
| 3617 (NS2A) | G | G | G | G | A | G |
| 3638 (NS2A) | G | L | G | L | A | L |
| 3677 (NS2A) | C | G | C | G | T | G |
| 3694 (NS2A) | C | T | C | T | T | I |
| 3710 (NS2A) | A | I | A | I | G | M |
| 3812 (NS2A) | A | T | A | T | G | T |
| 3813 (NS2A) | A | T | A | T | G | V |
| 3814 (NS2A) | C |  | C |  | T |  |
| 3815 (NS2A) | A |  | A |  | G |  |
| 3836 (NS2A) | C | L | C | L | T | L |
| 3863 (NS2A) | A | L | A | L | G | L |
| 3954 (NS2A) | T | S | T | S | C | P |
| 3990 (NS2A) | C | L | C | L | T | L |
| 4019 (NS2A) | T | C | T | C | C | C |
| 4074 (NS2A) | T | L | T | L | C | L |
| 4091 (NS2A) | G | L | G | L | A | L |
| 4151 (NS2B) | A | E | A | E | G | E |
| 4220 (NS2B) | C | A | T | A | T | A |
| 4262 (NS2B) | A | V | A | V | G | V |
| 4271 (NS2B) | C | G | C | G | T | G |
| 4304 (NS2B) | T | A | T | A | C | A |
| 4346 (NS2B) | T | S | T | S | C | S |
| 4367 (NS2B) | A | Q | A | Q | G | Q |
| 4373 (NS2B) | G | E | G | E | A | E |
| 4448 (NS2B) | G | T | G | T | A | T |
| 4479 (NS2B) | A | I | A | I | G | V |
| 4532 (NS3) | C | A | T | A | C | A |
| 4544 (NS3) | T | V | T | V | C | V |
| 4547 (NS3) | T | P | T | P | C | P |
| 4580 (NS3) | C | S | C | S | T | S |
| 4625 (NS3) | C | T | C | T | T | T |
| 4679 (NS3) | C | V | C | V | A | V |
| 4691 (NS3) | G | S | G | S | A | S |
| 4784 (NS3) | T | D | T | D | C | D |
| 4820 (NS3) | T | A | T | A | C | A |
| 4841 (NS3) | C | P | C | P | T | P |
| 4850 (NS3) | C | V | C | V | T | V |
| 4940 (NS3) | T | I | T | I | C | I |
| 4985 (NS3) | G | V | G | V | A | V |
| 5075 (NS3) | G | R | G | R | A | R |
| 5085 (NS3) | C | L | C | L | T | L |
| 5201 (NS3) | A | V | A | V | G | V |
| 5237 (NS3) | G | L | A | L | G | L |
| 5249 (NS3) | C | Y | C | Y | T | Y |
| 5252 (NS3) | A | Q | A | Q | G | Q |
| 5306 (NS3) | C | C | C | C | T | C |
| 5345 (NS3) | A | R | A | R | G | R |
| 5354 (NS3) | C | N | C | N | T | N |
| 5357 (NS3) | T | Y | T | Y | C | Y |
| 5369 (NS3) | A | V | A | V | G | V |
| 5399 (NS3) | C | S | C | S | T | S |
| 5435 (NS3) | A | V | A | V | G | V |
| 5489 (NS3) | G | T | G | T | A | T |
| 5534 (NS3) | A | R | A | R | G | R |
| 5537 (NS3) | G | E | G | E | A | E |
| 5612 (NS3) | T | P | T | P | C | P |
| 5651 (NS3) | G | R | G | R | A | R |
| 5702 (NS3) | A | E | A | E | G | E |
| 5717 (NS3) | G | K | G | K | A | K |
| 5768 (NS3) | T | A | T | A | C | A |
| 5771 (NS3) | C | N | C | N | T | N |
| 5849 (NS3) | C | I | C | I | T | I |
| 5861 (NS3) | C | P | C | P | T | P |
| 5888 (NS3) | C | A | C | A | T | A |
| 5891 (NS3) | A | Q | A | Q | G | Q |
| 5915 (NS3) | C | N | T | N | C | N |
| 6077 (NS3) | T | A | T | A | C | A |
| 6164 (NS3) | T | S | T | S | C | S |
| 6218 (NS3) | T | T | T | T | A | T |
| 6293 (NS3) | A | K | A | K | G | K |
| 6296 (NS3) | G | L | G | L | A | L |
| 6304 (NS3) | A | K | A | K | G | R |
| 6344 (NS3) | A | L | A | L | G | L |
| 6407 (NS4A) | C | I | C | I | T | I |
| 6422 (NS4A) | C | T | C | T | T | T |
| 6455 (NS4A) | C | D | C | D | T | D |
| 6464 (NS4A) | T | V | T | V | C | V |
| 6554 (NS4A) | C | A | C | A | T | A |
| 6602 (NS4A) | T | G | T | G | G | G |
| 6710 (NS4A) | G | L | G | L | A | L |
| 6809 (NS4A) | T | I | T | I | C | I |
| 6845 (NS4B) | A | I | A | I | T | I |
| 6912 (NS4B) | C | L | C | L | T | L |
| 6944 (NS4B) | G | A | G | A | A | A |
| 6992 (NS4B) | T | N | T | N | C | N |
| 7011 (NS4B) | T | L | T | L | C | L |
| 7067 (NS4B) | A | P | A | P | G | P |
| 7085 (NS4B) | T | L | T | L | C | L |
| 7153 (NS4B) | T | V | T | V | C | A |
| 7161 (NS4B) | C | L | C | L | T | L |
| 7238 (NS4B) | A | G | A | G | G | G |
| 7265 (NS4B) | G | G | A | G | G | G |
| 7274 (NS4B) | G | V | G | V | A | V |
| 7335 (NS4B) | T | L | T | L | C | L |
| 7409 (NS4B) | T | A | C | A | C | A |
| 7478 (NS4B) | T | S | T | S | C | S |
| 7511 (NS4B) | A | A | A | A | G | A |
| 7546 (NS4B) | C | A | C | A | T | V |
| 7556 (NS4B) | C | P | C | P | T | P |
| 7575 (NS5) | G | A | G | A | A | T |
| 7679 (NS5) | C | T | C | T | T | T |
| 7691 (NS5) | C | S | C | S | T | S |
| 7709 (NS5) | C | S | C | S | T | S |
| 7748 (NS5) | T | I | T | I | C | I |
| 7757 (NS5) | C | I | C | I | T | I |
| 7838 (NS5) | A | A | A | A | C | A |
| 7907 (NS5) | T | P | T | P | C | P |
| 8048 (NS5) | G | L | G | L | A | L |
| 8105 (NS5) | G | K | G | K | A | K |
| 8315 (NS5) | T | T | T | T | C | T |
| 8368 (NS5) | T | I | T | I | C | T |
| 8435 (NS5) | A | T | A | T | C | T |
| 8450 (NS5) | A | Q | A | Q | G | Q |
| 8513 (NS5) | G | A | G | A | A | A |
| 8534 (NS5) | G | V | G | V | A | V |
| 8537 (NS5) | G | V | G | V | A | V |
| 8564 (NS5) | A | V | A | V | G | V |
| 8570 (NS5) | G | P | G | P | A | P |
| 8597 (NS5) | C | D | C | D | T | D |
| 8654 (NS5) | G | P | G | P | A | P |
| 8666 (NS5) | A | P | A | P | C | P |
| 8705 (NS5) | A | L | A | L | G | L |
| 8804 (NS5) | A | Q | A | Q | G | Q |
| 8807 (NS5) | G | E | G | E | A | E |
| 8885 (NS5) | T | L | T | L | A | L |
| 8891 (NS5) | A | Q | A | Q | G | Q |
| 8918 (NS5) | C | Y | C | Y | T | Y |
| 8951 (NS5) | G | G | G | G | A | G |
| 8990 (NS5) | T | Y | T | Y | C | Y |
| 8999 (NS5) | A | L | A | L | G | L |
| 9050 (NS5) | T | H | T | H | C | H |
| 9068 (NS5) | C | N | C | N | T | N |
| 9113 (NS5) | C | Y | C | Y | T | Y |
| 9203 (NS5) | C | L | C | L | T | L |
| 9281 (NS5) | C | Y | C | Y | T | Y |
| 9356 (NS5) | G | Q | G | Q | A | Q |
| 9437 (NS5) | A | A | T | A | T | A |
| 9473 (NS5) | T | P | T | P | A | P |
| 9508 (NS5) | G | R | G | R | A | K |
| 9545 (NS5) | T | I | T | I | C | I |
| 9569 (NS5) | A | K | A | K | G | K |
| 9587 (NS5) | T | F | C | F | T | F |
| 9593 (NS5) | C | T | C | T | T | T |
| 9725 (NS5) | T | D | C | D | T | D |
| 9812 (NS5) | G | R | G | R | A | R |
| 9821 (NS5) | T | A | T | A | C | A |
| 9827 (NS5) | A | L | A | L | G | L |
| 9959 (NS5) | T | H | T | H | C | H |
| 9971 (NS5) | A | Q | A | Q | G | Q |
| 9989 (NS5) | C | D | C | D | T | D |
| 9998 (NS5) | G | K | G | K | A | K |
| 10031 (NS5) | C | P | C | P | T | P |
| 10039 (NS5) | T | I | T | I | C | T |
| 10070 (NS5) | T | D | T | D | C | D |
| 10130 (NS5) | T | S | T | S | C | S |
| 10169 (NS5) | T | T | T | T | C | T |
| 10181 (NS5) | T | N | T | N | C | N |
| 10211 (NS5) | T | Y | T | Y | C | Y |
| 10215 (NS5) | T | S | T | S | C | P |
| 10267 (3’UTR) | C | - | C | - | T | - |
| 10318 (3’UTR) | C | - | C | - | T | - |
| 10374 (3’UTR) | T | - | T | - | C | - |
| 10463 (3’UTR) | A | - | A | - | *Gap* | - |
| 10587^*^/8^#^ (3’UTR) | A | - | A | - | G | - |
| 10614^*^/5^#^  (3’UTR) | A | - | A | - | G | - |
